# A Benzenediamine Analog FC-99 Drives M2 Macrophage Polarization and Alleviates Lipopolysaccharide- (LPS-) Induced Liver Injury

**DOI:** 10.1155/2019/7823069

**Published:** 2019-07-31

**Authors:** Wei Gong, Haiyan Zhu, Li Lu, Yayi Hou, Huan Dou

**Affiliations:** ^1^Nanjing Key Laboratory of Pediatrics, Children's Hospital of Nanjing Medical University, 72 Guangzhou Road, Nanjing 210008, China; ^2^The State Key Laboratory of Pharmaceutical Biotechnology, Division of Immunology, Medical School, Nanjing University, Nanjing 210093, China; ^3^Department of Rheumatology and Immunology, Nanjing Drum Tower Hospital, The Affiliated Hospital of Nanjing University Medical School, Nanjing 210008, China; ^4^Jiangsu Key Laboratory of Molecular Medicine, Nanjing University, Nanjing 210093, China

## Abstract

Macrophages have variable functional phenotypes, high diversity, and plasticity and are involved in the pathogenesis of sepsis-induced liver injury. Alteration of macrophage polarization through activated (M1) macrophage to alternatively activated (M2) macrophage has emerged as a potential therapeutic strategy. This study was designed to explore the effect of a benzenediamine analog FC-99 on macrophage polarization in vitro and lipopolysaccharide- (LPS-) induced liver injury followed by the underlying mechanisms. For in vitro experiments, FC-99 inhibited M1-related macrophage factors and promoted M2-related markers induced by IL-4 in the mouse macrophage cell line RAW264.7. Moreover, FC-99-induced macrophages polarized to M2 phenotype which could be repressed by a PPAR-*γ* inhibitor but not STAT6 siRNA knockdown, indicating FC-99-induced M2 macrophage polarization through PPAR-*γ* rather than STAT6 signal. In LPS-induced septic mice, FC-99 pretreated mice displayed lower expression of M1 markers together with the increased M2 marker CD206 and improvement of liver injury. These findings illustrated that FC-99 could promote M2 macrophage polarization via PPAR-*γ* signaling and seemed to be a potential therapeutic candidate for inflammatory liver injury.

## 1. Introduction

Sepsis and subsequent multiple organ dysfunction syndrome (MODS) have become a major challenge in surgical critical care due to high morbidity and mortality in the absence of adequate treatment [1]. Liver dysfunction is an indicator of progression from sepsis to MODS. During the course of sepsis, the liver is considered one of the most frequently damaged organs and liver injury may occur at any stage [2, 3]. Therefore, effective prevention and treatment of sepsis-induced liver injury are urgently needed.

Macrophage is an important component in the first line of defensing against a hostile environment; it plays a key role in innate immunity and helps to initiate adaptive immune response [4]. Macrophages can be derived from circulating blood monocytes and exist as resident tissue-specific macrophages. As a professional phagocyte, macrophage displays multiple functions such as presenting antigens, repairing tissue, and modulating inflammation through various effector populations [5, 6]. The ability to alter the function of macrophages is called “polarization.” Depending on different environmental stimuli, macrophage mainly exists in two opposite activated populations, classically activated (M1) macrophage and alternatively activated (M2) macrophage [7]. The M1 phenotype is proinflammatory and secretes numerous inflammatory cytokines such as inducible nitric oxide synthases (iNOS), interleukin-6 (IL-6), and tumor necrosis factor (TNF-*α*) and is associated with microbicidal activation and tissue damage. Factors like interferon- (IFN-) *γ* or lipopolysaccharides (LPS) could induce the M1 macrophage polarization through NF-*κ*B pathways [8–10]. In contrast, the M2 macrophage can be induced by IL-4, IL-13, or monocyte colony-stimulating factor (M-CSF) and dampens inflammation by releasing anti-inflammatory factors such as IL-10. Besides, M2 is involved in homeostatic functions linked to tissue remodeling during inflammation or injury [11–13]. M2 increases the expression of a mannose receptor (also named CD206), arginase-1 (Arg-1), and chitinase-like proteins Ym-1 and Fizz [11]. Signal transducer and activator of transcription 6 (STAT6) and peroxisome proliferator-activated receptor- (PPAR-) *γ* were reported to control M2 polarization during IL-4/13 regulated signal pathways [14–17]. Studies have shown that the phenotypic switch of macrophage polarization was closely related to many diseases, especially those associated with inflammation. The balance between proinflammatory M1 and anti-inflammatory M2 serves to maintain homeostasis and defense, and a shift towards M2 macrophage may protect against inflammatory diseases as well as promote adverse tissue remodeling following injury [18, 19].

FC-99 (N1-[(4-methoxy)methyl],-4-methyl-1,2-benzenediamine) was a benzenediamine analog synthesized in our lab. In the previous studies, FC-99 displayed an anti-inflammatory effect and a therapeutic potential on experimental sepsis. It further inhibited the LPS-induced phosphorylation of NF-*κ*B, which were associated with M1 macrophage polarization [20, 21]. However, whether FC-99 directly modulates the M1/M2 polarization and the underlying mechanism of action remains unknown. Herein, we reported that macrophages treated with FC-99 acquired an M2 phenotype in vitro, and this induction was dependent on PPAR-*γ* but not STAT6. Furthermore, administration of FC-99 resulted in increased M2 macrophage polarization in the liver of septic mice under the premise of amelioration of liver injury. These data suggested that FC-99 could modulate macrophage polarization and is a possible therapeutic candidate for acute liver injury.

## 2. Materials and Methods

### 2.1. Reagents

FC-99, a pale brown crystalline powder with purity ≥ 98%, was prepared as previously described [21] and solubilized in dimethyl sulfoxide (DMSO) and diluted with saline (used in vivo) or PBS (used in vitro). Lipopolysaccharide (LPS) and PPAR-*γ* antagonist were purchased from Sigma-Aldrich (St. Louis, MO). Recombinant murine IL-4 was purchased from Peprotech (Rocky Hill, NJ). Anti-mouse CD16/32, anti-mouse APC-CD206, and its isotype control antibodies were purchased from BioLegend (San Diego, CA). Anti-phospho-STAT6 and total STAT6 were purchased from Cell Signaling Technology (Beverly, MA, USA), and anti-GAPDH and horseradish peroxidase (HRP) anti-rabbit IgG were purchased from Bioworld Technology Co. Ltd. (Nanjing, China). A mouse PPAR-*γ* ELISA kit was purchased from SenBeiJia Co. Ltd. (Nanjing, China).

### 2.2. Cells

Murine macrophage cell line RAW264.7, which was obtained from ATCC (American Type Culture Collection, Manassas, VA), was cultured in Dulbecco's modified Eagle's medium (Gibco, Grand Island, NY) with 100 U/ml penicillin G, 100 mg/ml streptomycin, and 10% fetal bovine serum (Gibco, Grand Island, NY). For macrophage polarization, RAW264.7 cells were treated with 10 ng/ml LPS for 12 h (M1) or 10 ng/ml IL-4 for 24 h (M2) according to the previous studies [22–24]. FC-99 with a dose of 50 *μ*M was used in the cell experiments, which showed no cytotoxicity as demonstrated in previous studies [21].

### 2.3. Reverse Transcription and Real-Time Quantitative PCR

Total RNA was extracted using a TRIzol reagent (Invitrogen, USA) as per the manufacturer's instructions and reverse-transcribed using a Revert Aid TM First Strand cDNA synthesis kit (Fermentas) in a total volume of 20 *μ*l. Next, real-time quantitative PCR (Q-PCR) was performed using SYBR Green PCR Master Mix (with Rox) (Invitrogen, USA) and a Step One Plus System (Applied Biosystems, Foster City, CA). The relative gene expression was normalized to GAPDH expression and assessed using the 2^-*ΔΔ*Ct^ method. The sequences of the primers were as follows: iNOS, 5′-CCAAGCCCTCACCTACTTCC-3′ (sense) and 5′-CTCTGAGGGCTGACACAAGG-3′ (antisense); Arg-1, 5′-CTCCAAGCCAAAGTCCTTAGAG-3′ (sense) and 5′-AGGAGCTGTCATTAGGGACATC-3′ (antisense); CD206, 5′-CTCTGTTCAGCTATTGGACGC-3′ (sense) and 5′-TGGCACTCCCAAACATAATTTGA-3′ (antisense); Ym-1, 5′-ATGAGTGGGTTGGTTATG-3′ (sense) and 5′-AAAGTAGATGTCAGAGGGA-3′ (antisense); and GAPDH, 5′-GGTGAAGGTCGGTGTGAACG-3′ (sense) and 5′-CTCGCTCCTGGAAGATGGTG-3′ (antisense).

### 2.4. Flow Cytometric Analysis

After treatment, cells were harvested and incubated with purified anti-CD16/32 (Fc blocker) for 10 min and then stained with anti-mouse APC-CD206 for 30 min at 4°C. APC-conjugated anti-Rat IgG*κ* was used as the isotype control. After incubation, cells were washed three times with phosphate-buffered saline (PBS) and then analyzed by a BD FACSCalibur flow cytometer (Bedford, MA).

### 2.5. Western Blot Analysis

Proteins were extracted, and 50 *μ*g proteins were electrophoresed on SDS polyacrylamide gels with Tris-glycine running buffer and transferred onto 0.45 mm PVDF membranes. After blocking with 5% (*w*/*v*) bovine serum albumin (BSA) in Tris-buffered saline (TBS) for 2 h at room temperature, the membrane was washed four times using TBST (TBS and 0.5% Tween-20) and then incubated with primary antibodies overnight at 4°C. After washing, the membrane was incubated with HRP-anti rabbit IgG for 2 h. Protein bands were visualized by ECL Plus western blotting detection reagents (Millipore, USA). GAPDH was used as an internal control. Each blot was a representative of three independent experiments, and band intensity was measured using ImageJ software.

### 2.6. Small Interfering RNA (siRNA) Knockdown

For silencing STAT6 (NM_009284.2), siRNA knockdown was performed in RAW264.7 cells using siRNA duplexes purchased from RiboBio (Guangzhou, China). The negative control siRNA (scrambled siRNA) was provided by RiboBio. The two independent oligonucleotides designed for STAT6 were as follows: (1) 5′-CCAAGACAACAACGCCAAA dTdT-3′ and (2) 5′-GCUGAUCAUUGGCUUUAUU dTdT-3′. The siRNA fragments were transfected into RAW264.7 cells by electroporation using the Cell Line Nucleofector Kit V (Lonza) and program D-32 of an AmaxaNucleofector (Amaxa, Cologne, Germany) according to the manufacturer's protocol. Cells were then recovered for 48 h, and the silencing effect was detected by Q-PCR.

### 2.7. Enzyme-Linked Immunosorbent Assay (ELISA)

For intracellular PPAR-*γ* measurement, RAW264.7 cells were pretreated with or without FC-99 for 24 h. After incubation, proteins were extracted and the protein concentration of each sample was determined by the BCA protein assay kit (Pierce Chemical, Rockford, IL). An equivalent amount of proteins from each treated group was measured using a PPAR-*γ* ELISA kit according to the manufacturer's instructions.

### 2.8. Animals

Specific pathogen-free (SPF) male BALB/c mice (aged 8–10 weeks) were purchased from Model Animal Genetics Research Center of Nanjing University (Nanjing, China). Animal welfare and experimental procedures were in strict accordance with the Research Ethics Committee of Nanjing University. An LPS-induced septic model was established and treated with the same protocol as previously described [21]. For FC-99 administration, mice were pretreated with FC-99 (100 mg/kg, i.p. injection) 2 h prior to LPS challenge. After 24 h, mice were anaesthetized, bled, and killed. Serum and liver tissue were collected for alanine aminotransferase (ALT) and aspartate aminotransferase (AST) measurements (Beckman Coulter, CA, USA), hematoxylin and eosin (H&E) staining (Google Biotech, Wuhan, China), and macrophage phenotype analysis. The degrees of liver injury were scored with a scale of 0-3 in a double-blind fashion, and at least three slides were studied from each specimen.

### 2.9. Immunofluorescence (IF)

Frozen sections of the liver tissue from mice were obtained. After being washed three times with phosphate-buffered saline (PBS), the sections were blocked at 15 to 25°C for 30 min. IF was conducted as previously described [25].

### 2.10. Statistical Analysis

Each experiment was repeated at least three times. All data were expressed as the mean ± SEM and analyzed by *t*-test for the independent group. A statistical significance was set to *p* < 0.05. All calculations were performed with GraphPad Prism (GraphPad Software, San Diego, CA, USA).

## 3. Results

### 3.1. FC-99 Inhibits the Expression of M1 Phenotypes Induced by LPS and Promotes M2 Marker Expression Induced by IL-4

**Figure 1:**
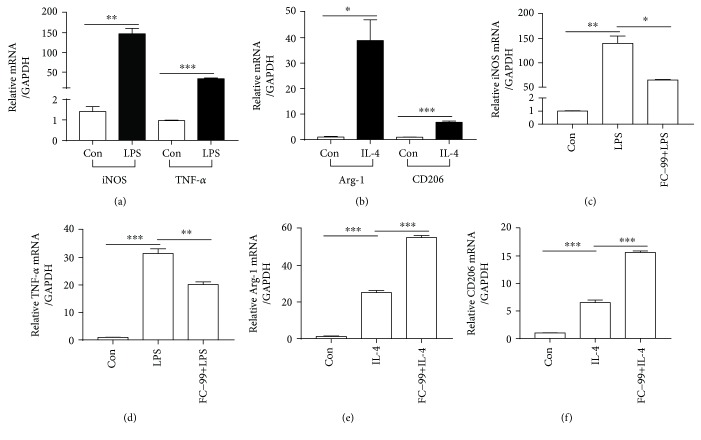
FC-99 inhibited the expression of M1 phenotypes induced by LPS and promoted M2 marker expression induced by IL-4. RAW264.7 cells were cultured in 10 ng/ml LPS for 12 h to establish M1 or in 10 ng/ml IL-4 for 24 h to establish M2 macrophage, respectively. (a, b) For the identification of M1 and M2 phenotypes, M1 (iNOS and TNF-*α* (a)) and M2 markers (Arg-1 and CD206 (b)) were tested using Q-PCR. (c, d) RAW264.7 cells were stimulated by LPS with or without FC-99 (50 *μ*M) for 12 h; mRNA levels of iNOS (c) and TNF-*α* (d) were determined by Q-PCR. (e, f) RAW264.7 cells were stimulated by IL-4 with or without FC-99 (50 *μ*M) for 24 h; mRNA levels of Arg-1 (e) and CD206 (f) were determined by Q-PCR. Data were presented as the mean ± SEM. ^∗^*p* < 0.05, ^∗∗^*p* < 0.01, and ^∗∗∗^*p* < 0.001 vs. the indicated group.

We first performed cellular phenotype identification using RAW264.7 cells under different stimulators. As shown in Figures 1(a) and 1(b), M1 markers iNOS and TNF-*α* were highly upregulated after LPS stimulation, while M2 polarization was induced by IL-4 in which the relative markers Arg-1 and CD206 were significantly enhanced. Next, we tested the effect of FC-99 on macrophage polarization. FC-99 significantly suppressed the levels of iNOS and TNF-*α* induced by LPS (Figures 1(c) and 1(d)), exhibiting an anti-inflammatory activity as previously described [20]. In contrast, cotreatment with IL-4 and FC-99 resulted in higher levels of Arg-1 and CD206 than IL-4 treatment alone (Figures 1(e) and 1(f)). These results suggested that FC-99 may promote M2 macrophage activation and inhibit M1 polarization.

### 3.2. FC-99 Directly Modulates M2 Macrophage Polarization In Vitro

**Figure 2:**
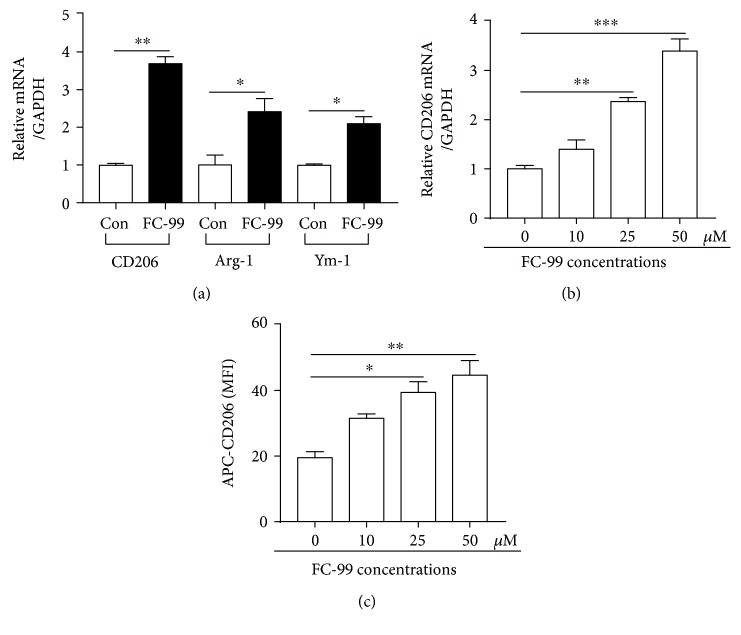
FC-99 directly modulated M2 macrophage polarization. RAW264.7 cells were incubated with FC-99 (50 *μ*M) for 24 h; (a) mRNA levels of CD206, Arg-1, and Ym-1 were assessed by Q-PCR. (b, c) RAW264.7 cells were incubated with the indicated concentrations of FC-99 for 24 h, and CD206 expression was measured by Q-PCR (b) and flow cytometry (c). Data were presented as the mean ± SEM. ^∗^*p* < 0.05, ^∗∗^*p* < 0.01, and ^∗∗∗^*p* < 0.001 vs. the indicated group.

To further determine the effect of FC-99 on macrophage polarization, RAW264.7 cells were treated with FC-99 alone for 24 h. As shown in Figure 2(a), FC-99 significantly increased the expressions of M2 markers CD206, Arg-1, and Ym-1, of which CD206 showed the highest expression. Hence, CD206 was chosen as the measurement indicator for the subsequent experiments. As shown in Figures 2(b) and 2(c), FC-99 increased CD206 expression in a dose-dependent manner, as indicated by mRNA and protein levels.

### 3.3. FC-99-Induced M2 Polarization Is Independent of STAT6

**Figure 3:**
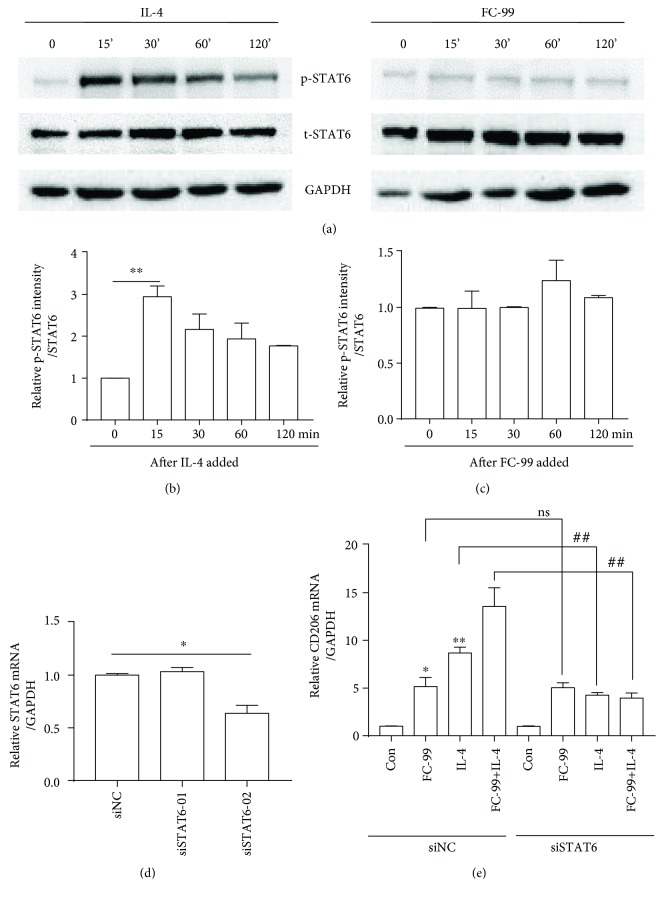
Induction of FC-99 on M2 polarization was independent of STAT6. (a–c) RAW264.7 cells were cultured with IL-4 (10 ng/ml) or FC-99 (50 *μ*M) for the indicated time points; levels of STAT6 phosphorylation (p-STAT6), total STAT6 (t-STAT6), and GAPDH were tested by Western blot. (b) and (c) were the relative intensity analysis of p-STAT6 using STAT6 as the internal control. (d) The silencing effect of siRNA for STAT6 was detected using Q-PCR. Data were presented as the mean ± SEM. ^∗^*p* < 0.05, ^∗∗^*p* < 0.01, and ^∗∗∗^*p* < 0.001 vs. the indicated group. (e) RAW264.7 cells were transfected with control siRNA (si-NC) or siRNA targeting STAT6 (si-STAT6) for 48 h, and then cells were treated with FC-99 (50 *μ*M), IL-4 (10 ng/ml), or both, respectively, for another 24 h. The mRNA level of CD206 was measured by Q-PCR. Data were presented as the mean ± SEM. ^∗^*p* < 0.05 and ^∗∗^*p* < 0.01 vs. Con; ^##^*p* < 0.01 vs. the indicated group.

Next, we investigated the underlying molecular mechanism of FC-99 in macrophage polarization. STAT6 is an important transcription factor involved in an IL-4-induced classical pathway during macrophage polarization [17]. As a general mechanism, STAT6 phosphorylation was induced by IL-4 at 15 min (Figures 3(a) (left panel) and 3(b)). Unlike IL-4, FC-99 could not induce the phosphorylation of STAT6 (Figures 3(a) (right panel) and 3(c)). To further investigate whether the STAT6 pathway was involved in FC-99-induced M2 polarization, siRNA interference of STAT6 was used and the siSTAT6-02 fragment was chosen because of its significant reduction on the mRNA levels of STAT6 (Figure 3(d)). As shown in Figure 3(e), STAT6 knockdown suppressed the expression of CD206 induced by IL-4 and IL-4-FC-99 combination but had no effect on FC-99 treated alone. These results suggested that STAT6 was not necessary for FC-99-induced CD206 expression.

### 3.4. FC-99 Mediates M2 Macrophage Polarization via PPAR-*γ*

**Figure 4:**
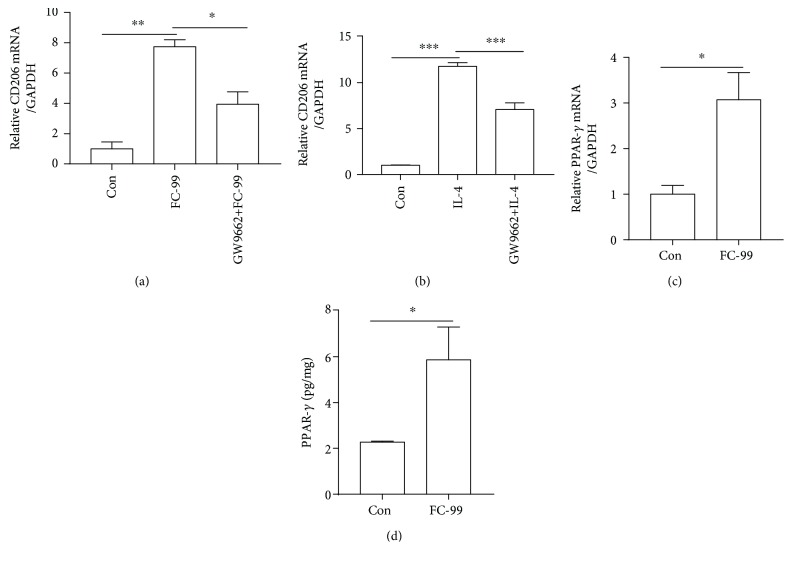
FC-99 mediated M2 macrophage polarization via PPAR-*γ*. (a, b) RAW264.7 cells were pretreated with GW9662 at 1 *μ*M for 2 h before IL-4 or FC-99 treatment for another 24 h, and the mRNA expression of CD206 was measured by Q-PCR. (c, d) RAW264.7 cells were treated with FC-99 (50 *μ*M) for 24 h, the expression of PPAR-*γ* in the mRNA level was tested by Q-PCR (c), and the protein level in cell extracts (1 mg protein) was assessed by ELISA (d). Data were presented as the mean ± SEM. ^∗^*p* < 0.05, ^∗∗^*p* < 0.01, and ^∗∗∗^*p* < 0.001 vs. the indicated group.

PPAR-*γ*, a ligand-activated transcriptional factor, is a member of the nuclear hormone receptor family and plays an essential role during macrophage alternative activation [14]. Therefore, we tested the role of PPAR-*γ* in the FC-99-induced M2 activation using a PPAR-*γ*-specific antagonist GW9662. The expression of CD206 was significantly increased, respectively, in FC-99 and IL-4 treatment, while both of them were disrupted by GW9662 pretreatment (Figures 4(a) and 4(b)); moreover, upon stimulation with FC-99, PPAR-*γ* expression was upregulated at the mRNA level, and consistently, the intracellular content of PPAR-*γ* protein was also increased and tested by ELISA (Figures 4(c) and 4(d)). The above data indicated that FC-99 mediated M2 macrophage polarization via PPAR-*γ* signaling.

### 3.5. FC-99 Facilitates the Phenotype Shift from M1 to M2 in the Liver of Septic Mice

**Figure 5:**
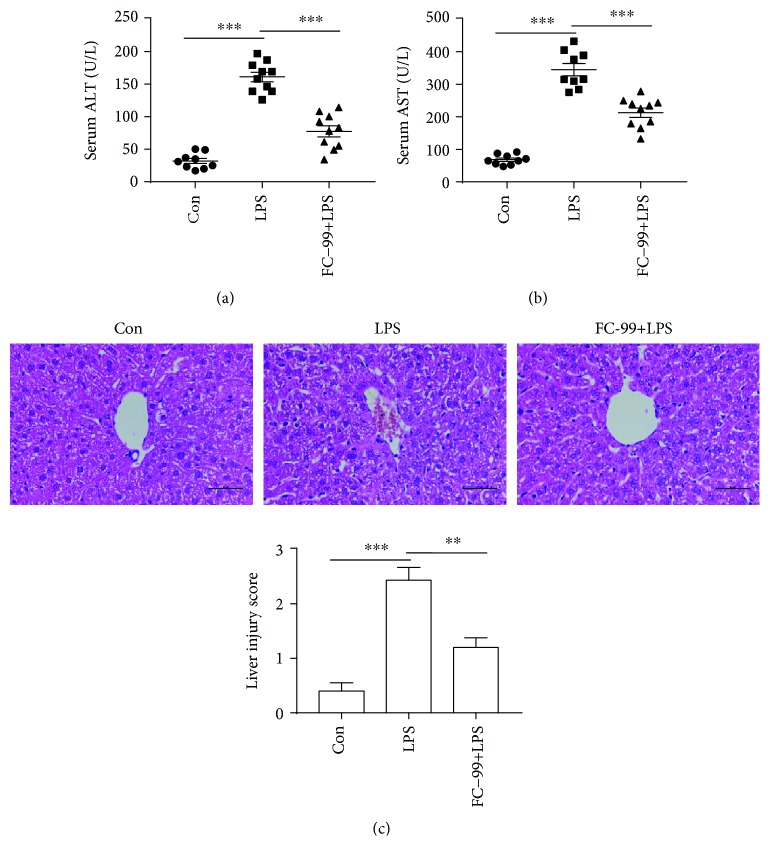
FC-99 alleviated LPS-induced mouse liver injury. Mice were pretreated with FC-99 (100 mg/kg, i.p. injection) 2 h prior to LPS challenge. (a, b) Serum concentrations of ALT and AST were assessed 24 h after LPS (10 mg/kg, i.p. injection) treatment. (c) Representative images of liver H&E staining. Scale bar: 50 *μ*m. The histogram shows the liver injury scores based on the H&E staining images. Data were presented as the mean ± SEM. ^∗∗^*p* < 0.01 and ^∗∗∗^*p* < 0.001 vs. the indicated group.

**Figure 6:**
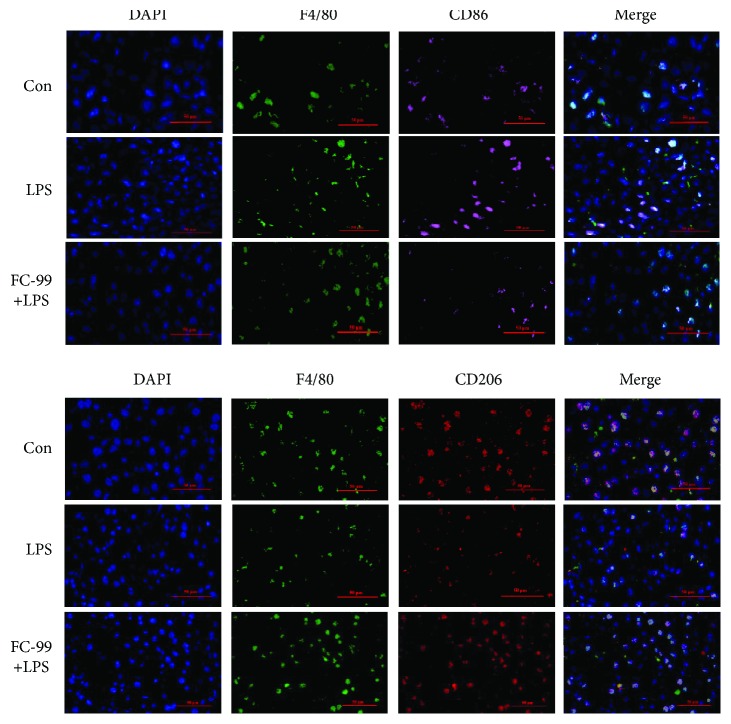
FC-99 facilitated the phenotype shift from M1 to M2 in the liver of septic mice. Mice were pretreated with FC-99 (100 mg/kg, i.p. injection) 2 h prior to LPS challenge (10 mg/kg, i.p. injection). After 24 h, the F4/80, CD86, and CD206 in the liver tissues were detected by immunofluorescence. Scale bar: 50 *μ*m.

To demonstrate the effect of FC-99 on macrophage polarization in vivo, the LPS-induced septic mouse model was utilized. Firstly, we confirmed the therapeutic effect of FC-99 on liver injury in the septic mice and found that the levels of the indicators of hepatic dysfunction serum alanine transaminase (ALT) and aspartate aminotransferase (AST) were significantly reduced by FC-99 treatment (Figures 5(a) and 5(b)). The liver histological analysis by H&E staining showed the ameliorated pathological changes including inflammatory cell infiltration, congestion, necrosis, and degeneration in septic mice administered with FC-99, and similarly, the liver injury score of FC-99-treated mice was markedly decreased compared with the LPS-only treatment group (Figure 5(c)). Furthermore, we confirmed whether the macrophage phenotype changed in a mouse liver after FC-99 treatment. Macrophage was labeled with F4/80 [26]. CD86, which was a proinflammatory cell surface factor, was served as the M1 marker in tissues [27]. As shown in Figure 6, CD86 was significantly decreased while the M2 marker CD206 was upregulated in the liver of septic mice after FC-99 treatment, suggesting that FC-99 may inhibit M1 and promote M2 polarization in the liver of a septic model.

## 4. Discussion

Sepsis is a serious infection with systemic inflammatory response and increased mortality rates in patients. During the course of sepsis, excessive inflammatory cytokines are released that lead to the recruitment of leukocytes, multiple organ damage, and especially acute liver injury. The liver is one of the first and the most seriously damaged organs during sepsis [2, 28–30]. Recent studies showed a central role for macrophages in the pathophysiology of acute hepatic diseases in animal models and patients [31, 32]. Macrophages are characterized by high diversity, plasticity, and different functional phenotypes that are involved in various pathological processes. During inflammation, numerous M2 macrophages contributed to the control of inflammation and repair of damaged tissues [33]. Correspondingly, strategy on the regulation of macrophage polarization was proposed as a potential therapy for hepatic inflammatory diseases.

In our previous studies, we synthesized a novel benzenediamine derivative FC-99 and tested its biological activity both in vitro and in vivo. FC-99 was identified as an inflammatory inhibitor that suppressed macrophage inflammatory response induced by LPS through the NF-*κ*B signal pathway [21]. Since FC-99 was anti-inflammatory and suppressed LPS-induced NF-*κ*B activation, which was associated with M1 polarization [34], the present study was designed to investigate the role of FC-99 in macrophage polarization. The results showed that FC-99 suppressed LPS-induced M1 polarization in RAW264.7 cells and induced the expression of M2 markers such as CD206, Arg-1, and Ym-1 in the absence of IL-4. Besides, FC-99 demonstrated a dose-dependent increase in the CD206 protein level. These observations suggested that RAW264.7 cells treated with FC-99 acquired an anti-inflammatory M2 phenotype. Consistently, in the liver, from FC-99-treated septic mice, a switch toward M2-like phenotype in vivo was displayed.

The molecular mechanism of macrophage polarization is not clearly understood. Previous studies indicated that activation of STAT6 induced by IL-4 or IL-13 promoted expression of M2-related gene expression [17]. However, phosphorylation of STAT6 was not observed in FC-99-treated macrophage, and this phenomenon was in accordance with our previous studies which displayed the inhibitory effect of FC-99 on kinase phosphorylation activity [21]. Meanwhile, STAT6 siRNA did not alter the induction of FC-99 on CD206 expression, indicating that the effect of FC-99 on M2 polarization was not dependent on STAT6. PPAR-*γ* is a member of the nuclear receptor superfamily of ligand-dependent transcription factors, which was first identified in adipose tissue, and participated in lipogenesis and glucose metabolism. Subsequent studies also indicated a potential role of PPAR-*γ* in M2 polarization [14, 35]. We found that PPAR-*γ* was necessary for FC-99-induced M2 activation, and this effect was significantly inhibited when the activity of PPAR-*γ* was inhibited by a specific antagonist GW9662. Benzimidazole derivatives were demonstrated to be associated with the transcription and activation of PPAR-*γ* [36]. Since FC-99 has a similar chemical group as these derivatives [21], we speculated that FC-99 might directly induce PPAR-*γ*. The specific underlying mechanism needs further investigation.

In summary, FC-99 suppressed the M1-related inflammatory response and promoted a switch to an alternatively activated M2 phenotype. This effect on macrophage polarization occurred directly through PPAR-*γ* and did not rely on the classical STAT6 pathway. Furthermore, FC-99 also reprogramed macrophages toward M2 phenotype in the liver of sepsis-induced mice, which might be another potential mechanism underlying FC-99 therapy on the liver from the LPS-induced mouse model. Collectively, FC-99 represented a novel therapy of altering macrophage polarization in LPS-induced liver injury.

## Data Availability

The data used to support the findings of this study are available from the corresponding authors upon request.
